# Tree Weights of *Avicennia germinans* in Mangrove Ecosystems Along the Guyana Coastline

**DOI:** 10.1002/pei3.70071

**Published:** 2025-07-15

**Authors:** Sabrina Dookie, Sirpaul Jaikishun, Abdullah Adil Ansari

**Affiliations:** ^1^ Department of Biology University of Guyana Georgetown Guyana; ^2^ Faculty of Natural Sciences University of Guyana Georgetown Guyana

**Keywords:** *Avicennia germinans*, biomass allocation, climate change, disturbances, ecosystems, Guyana, mangroves, trees

## Abstract

Mangroves are known as highly functional and productive ecosystems despite the numerous human and environmental disturbances they face continuously. These disturbances are known to affect their ecosystem states as well as their biomass allocation in their roots, trunks, stems, and leaves. We utilized a combination of plotless sampling methods and established common allometric equations to examine and compare the aboveground, trunk, and root weights of over 600 *Avicennia germinans* trees found along the Guyana coastline in natural, degraded, and restored ecosystems. Our results highlighted that while the restored ecosystems possessed taller trees with greater densities, the natural ecosystems possessed trees with greater aboveground (54396.24 kg/ha), trunk (19127.08 kg/ha), and root weights (20984.44 kg/ha) due to greater diameter at breast height values (> 30–40 cm). Furthermore, positive correlation coefficients (0.97 < *r*_s_ < 1.00) and regression values (*p* < 0.05) yielded compelling evidence in favor of the relationship between biomass allocation through tree organ weights and ecosystem types. Our findings support the notion that the composition and magnitude of disturbances within an ecosystem may affect mangrove tree biomass, hence influencing the net primary productivity of mangrove forests over time. This may have implications for their ability to accumulate and allocate biomass, as well as store carbon in the future. As such, the proactive conservation of existing mangrove forests is crucial for sustaining their productivity and viability, as well as augmenting their significance in biogeochemical cycles and their role in mitigating climate change.

## 1 Introduction

Mangrove forests, consisting of intricate bionetworks of halophytic plants and shrubs found in tropical and subtropical regions, dwell in two worlds. They are positioned at the interface of terrestrial and marine ecosystems and have garnered widespread attention due to their ecological value, resilience, and significant contributions to these ecosystems (Long et al. 2025; Rog et al. 2016; Giri et al. 2011). Popularly referred to as “blue carbon forests,” mangroves provide numerous ecological goods and services locally and globally to many coastal populations (Ermgassen et al. 2020). In addition, these habitats provide several benefits that contribute to poverty alleviation and food security, including sustainable food sources and the provision of raw materials such as charcoal, non‐timber forest goods, lumber, and fish/shellfish (Dookie et al. 2024a). Mangroves play a crucial role in supporting and regulating essential processes, including the hydrodynamic attenuation of waves and sediment stabilization (Kathiresan 2021; Friess 2016). Recently, there has been a growing emphasis on carbon sequestration as a vital regulating ecosystem service in mangroves, seen as a potential strategy to reduce a portion of human‐induced carbon dioxide emissions (Mishra et al. 2025; Inoue 2018). People often overlook the diverse cultural ecosystem services that mangroves offer to communities. This spectrum includes both tangible aspects, like tourism and education, as well as abstract qualities, such as religious, traditional, and cultural aesthetics (Zurba et al. 2022). However, mangroves are currently experiencing considerable declines due to human activities and are further threatened by global climate change (Hagger et al. 2022). Mangroves have experienced substantial damage and deforestation, resulting in a loss of 20%–35% of global mangrove extent over the past 50 years (Polidoro et al. 2010). Additionally, IUCN (2024) has reported that approximately 50% of mangrove ecosystems are at risk of collapse, representing 50% of the total global coverage. In the twentieth century, the primary drivers of global mangrove losses are derived from both political and economic aspects of development, including extensive forest clearing and the exploitation of these ecosystems for aquaculture and agriculture practices, timber, and raw materials, alongside the swift growth of coastal populations and urban development (Goldberg et al. 2020; Thomas et al. 2017).

Our study focuses on the mangroves of Guyana's coastline—a 458‐km stretch which consists of approximately 33,277 ha of mangroves occupying cheniers, mudflats, and sandy beaches adjacent to a swampy coastal plain (Dookie et al. 2022b). The coastline of Guyana features three prominent species of mangroves arranged in a specific zonation pattern: *Avicennia germinans* (L.) L. (black mangrove) (coast), *Rhizophora mangle* L. (red mangrove) (not coast), and *Laguncularia racemosa* (L.) C.F.Gaertn. (white mangrove) (coast) (Jaikishun et al. 2017). The mangroves along Guyana's fragile coastline are recognized for offering various essential ecological goods and services, such as lumber production, vital coastal protection, flooding prevention, sea defense, and the sustainability of integrated aquaculture and wildlife in the coastal environment (Guyana Forestry Commission 2017). These distinct forests provide a habitat for a variety of plants and animals that rely on mangroves for nesting sites during high tides, for shelter, and as a source of nourishment (Dookram et al. 2017). Moreover, mangrove trees settled along the Guyana coastline break down contaminants, significantly contributing to carbon sequestration and yielding various products such as charcoal, medicine, honey, tannin, and fodder (Dookie et al. 2022a). When compared to other South American nations such as Brazil, Suriname, and Ecuador, the decline of mangroves throughout the Guyana coastline is mainly due to human activities such as increased land use for the development of urban areas, agriculture, aquaculture, enhanced infrastructure, and overexploitation (NAREI 2019).

In May 2015, ExxonMobil reported the discovery of substantial quantities of oil in offshore regions of Guyana. There is a compelling argument that the recent oil discoveries could substantially enhance Guyana's gross domestic product (McDonald and Üngör 2021); however, there are potential environmental repercussions that may arise in the future, particularly concerning the coastal forests of Guyana. This prompts a re‐evaluation of the existing functionality and productivity of mangroves, alongside their capacity for “blue carbon” sequestration and the significant role they fulfill in mitigating climate change. A method to achieve this involves evaluating the weight, or “biomass,” of the trees present in these forests. Mangroves, thriving in muddy and anaerobic soils subjected to tidal inundation, exhibit a distinctive pattern of biomass allocation (Komiyama et al. 2005). A multitude of research endeavors has underscored the significance of quantifying tree weight or biomass, which is essential for precise evaluations of carbon storage potential, monitoring of forest vitality, regulation of timber extraction, and understanding the extensive influence of forest ecosystems on climate change (Abdul‐Hamid et al. 2022; Komiyama et al. 2016). The weight of a tree serves as a direct indicator of its capacity to sequester carbon dioxide from the environment. This facilitates more accurate assessments of carbon sequestration, carbon credit allocations, and informs forest management strategies that prioritize biomass over just tree quantity (Indrayani et al. 2021; Jones et al. 2020).

The weight of trees is generally influenced by the composition of mangrove communities, the availability of nutrients, and hydro‐geomorphic contexts characterized by varying sediment surface elevations. The climatic conditions and hydro‐geomorphic settings present within the mangrove environment may serve as critical determinants influencing primary productivity and plant biomass (Wang et al. 2021). With the growing encroachment of urban development on mangrove forests, these ecosystems face a multitude of additional pressures resulting from the urban environment. The increasing concentration of contaminants, including sewage, runoff, and industrial wastewater, poses significant threats to their development and survival rates (Szafranski and Granek 2023). Furthermore, it has been documented that environmental pollution, heightened levels of disturbance, and climate change significantly impact the development and productivity of mangroves (Nguyen 2024), thereby modifying their weights. Pollution also greatly reduces the structural weight of mangroves, both above and below ground, through growth inhibitions, damage to their roots, and increased stress on their overall structure through the accumulation of toxic substances in their tissues. This cascade of effects ultimately results in reduced plant vitality and increased mortality rates (Sivan et al. 2025; Szafranski and Granek 2023; Meng et al. 2021; Srikanth et al. 2015).

Our study applies the use of allometry, an effective tool for estimating tree weight based on independent variables including trunk diameter and height, which can be measured in the natural environment. An allometric relationship can be anticipated when the structural makeup of a tree body is grounded in biological or physical concepts (Yao et al. 2024; Abdul‐Hamid et al. 2022; Indrayani et al. 2021; Komiyama et al. 2005). Biomass allometric models facilitate the estimation of tree weight based on tree size, employing the principles of allometric scaling theory. This theory posits a general power‐law connection between tree biomass and tree diameter, characterized by a constant scaling exponent (Chave et al. 2005). The allometric scaling theory's effectiveness in determining biomass in tropical forests may be questionable in certain instances; however, the principle that body size, specifically tree size, significantly accounts for the structural, functional, and ecological traits of an organism, such as tree biomass, remains valid (Vieilledent et al. 2012).

The potential uses and effective utilization of allometric equations in various mangrove ecosystems remain inadequately understood and quantified, especially in the tropical coastal forests of South America. Furthermore, the impact of prevailing conditions on the weight of mangrove vegetation across different mangrove ecosystem types is not well understood due to a dearth of existing studies on the subject matter. Within this contention, we hypothesized (H_0_) that there are no statistically significant differences in the tree organ weights of *A. germinans* found in the natural, degraded, and restored ecosystems. Our study, therefore, aims to (i) determine the tree trunk weight, aboveground weight, and root weight of *A. germinans* trees using established common allometric equations and (ii) compare the weight of *A. germinans* tree organs in natural, degraded, and restored mangrove ecosystems. In our perception, we believe that this study holds significance due to the growing role of mangroves in global climate change mitigation through carbon storage, as well as the substantial emissions resulting from their destruction. Examining the influence of various ecosystem types on the biomass of mangrove vegetation contributes to more accurate estimates for carbon financing and accounting methods, which depend on capturing variability to enhance the reliability of both regional and global carbon stock assessments.

## 2 Materials and Methods

### 2.1 Description of Study Sites

**FIGURE 1:**
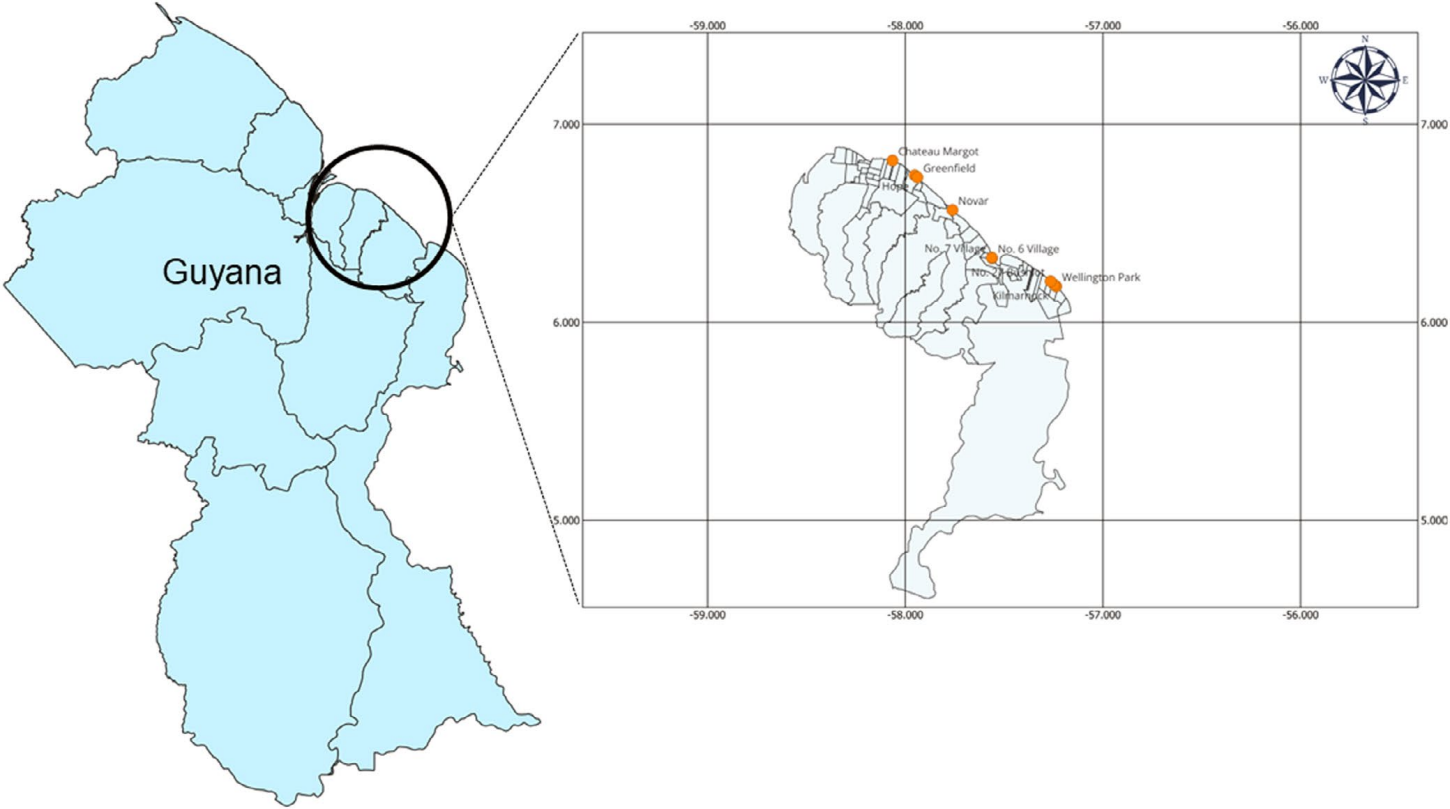
Map of Guyana showing the nine study sites along the coastline.

Our study was conducted in nine fringe mangrove ecosystems along the coastal regions of Guyana, in Administrative Regions 4–6. Among the nine selected locations, three were identified as natural mangrove ecosystems: Novar, Number 27 Village, and Kilmarnock. Additionally, three locations were identified as degraded: Hope, Greenfield, and Wellington Park. In contrast, the remaining three sites—Chateau Margot, Number 6 Village, and Number 7 Village—were classified as restored ecosystems (Figure 1). Data collection occurred in July 2023, coinciding with the wet season. The restored mangrove ecosystems included trees that have not reached full maturity, often referred to as “juvenile forests.” The Guyana Mangrove Restoration Project, initiated by the Ministry of Agriculture, focused on the intentional restoration of these mangrove ecosystems over a decade ago. The natural mangrove ecosystems display specific characteristics, such as the presence of mature trees that have persevered over time. These environments are characterized by low levels of disturbance (Dookie et al. 2023a) (Table 1). Conversely, the degraded mangrove ecosystems have experienced a substantial decline, primarily due to both natural events and human activities involving insufficient waste management and noticeable pollution in both the vegetated regions and beyond. These activities display substantially greater levels in contrast to the other two ecosystem types. Noteworthily, anthropogenic impacts included the building of infrastructure, poor management of waste, overgrazing of cattle, aquaculture and fishing, and cutting/burning of vegetation. The air temperatures of these coastline forests generally vary from 24°C to 28°C in the wet season and 29°C–31°C in the dry season (Dookie et al. 2023b, 2024a). While the soils in restored ecosystems consist primarily of heavy clay, the soils in degraded ecosystems generally show a broad spectrum of compositions, from compact loamy sands to less dense sands. The soils found in natural ecosystems often show a variety of textures, from sandy clay to medium clay (Dookie et al. 2024b).

**TABLE 1:** Types and extent of disturbances in the natural, degraded, and restored mangrove ecosystems studied along the Guyana coastline.

Ecosystem type			Degraded	Restored	Natural
			Extent of disturbances		
Types of disturbances	Natural	Plant infestation	+	+++	++++
		Storms/tides	+++++	++	++
		Erosion	++++++	+	++
		Insect infestation	+	+++	++++
		*Sargassum* invasion	++++	++	++
	Anthropogenic	Bark stripping	++++	+	+
		Grazing	++++	++++	++
		Cutting	++++++	+	++
		Sand mining	+++++	+	+
		Burning	++++	+	++
		Fishing activities	+++++	++++	+++
		Garbage dumping	+++++	+	+++
		Marine & coastal litter	+++++	++	+++
		Infrastructure development	+++++	++	++
		Lumbering (sawdust)	+++++	+	++
		Seashell mining	+++++	+	++

*Note:* +very low, ++low, +++average, ++++high, +++++very high.

### 2.2 Experimental Design

#### 2.2.1 Sampling of Trees Using the Point Centered Quarter Method (PCQM)

**FIGURE 2:**
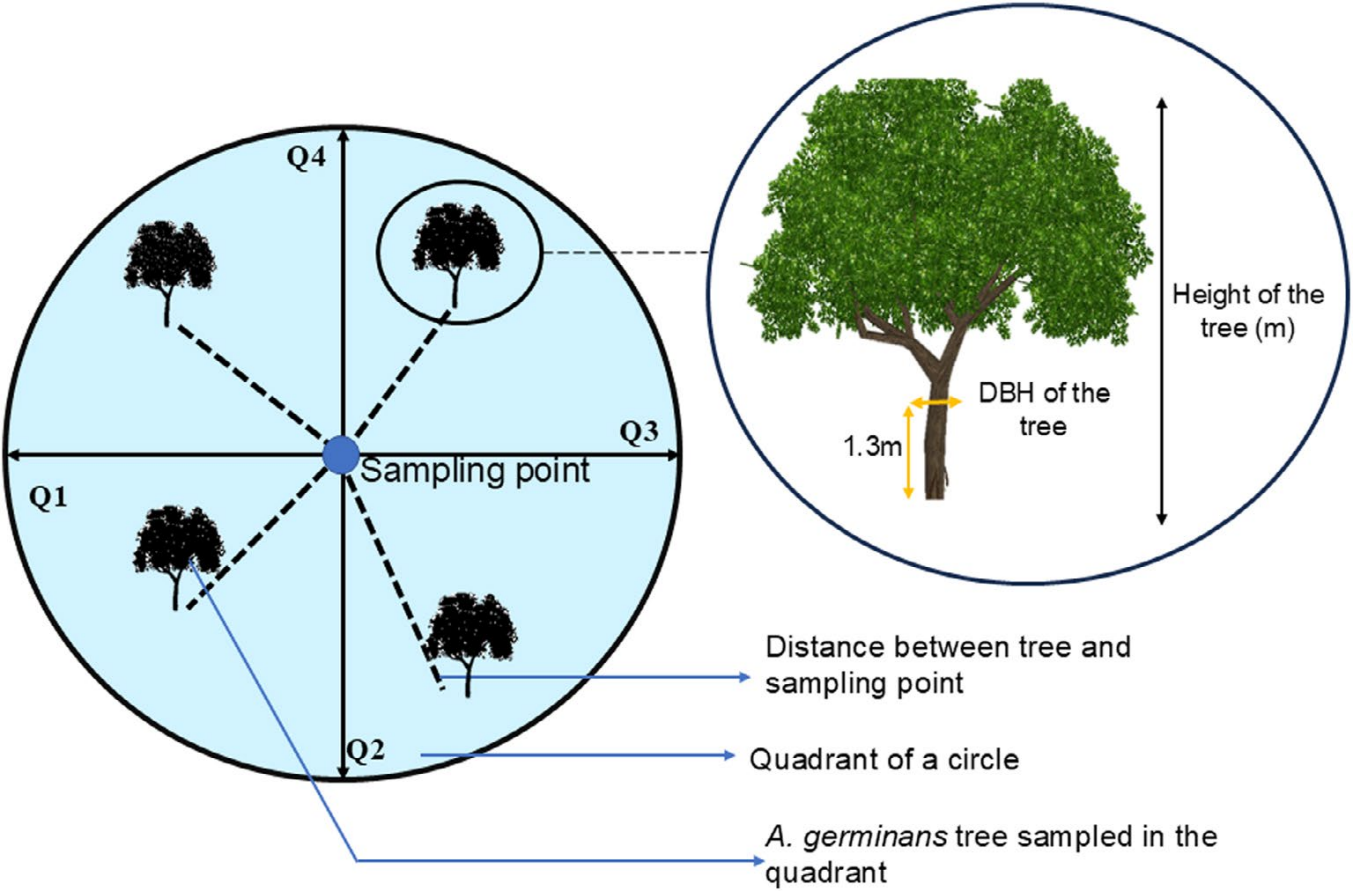
Sampling of *A. germinans* trees using the point centered quarter method (PCQM). NB: DBH, diameter at breast height.

The PCQM was used to sample over 600 *A. germinans* trees from the 9 study sites (Cottam and Curtis 1956). *Avicennia germinans* (commonly referred to as black mangrove, “Cruda bush,” or *A. germinans* ) typically reaches heights of 20–25 m and is distinguished by its dark trunk, robust, leathery, dark green foliage featuring a grayish underside, and, most notably, its distinctive pneumatophores (Dookie et al. 2023a; Hopkinson et al. 2022). We established a 250 m line transect 10 m from the shoreline of each sampling area. The line transect was then divided into 25 points at 10 m intervals. At each point, a circle of radius 10 m was constructed and divided into four quadrants—North, South, East, and West. Within each quadrant, the *A. germinans* tree nearest to the sampling point was selected after which the following measurements were taken following the method adopted by Jaikishun et al. (2017) and Dookie et al. (2023a): (1) the distance of the tree away from the sampling point (in m), (2) the estimated height (measured with a Nikon Forestry Pro II rangefinder) (in m), and (3) the diameter at breast height (measured using a DBH tape and taken at 1.3 m from the base of the tree) (Figure 2).

After data collection, the tree datasets were compiled according to each ecosystem type and were categorized into the following diameter classes > 5–10, > 10–20, > 20–30, > 30–40, and > 40 cm. Furthermore, the density (individuals/ha), basal area (m^2^/ha), and a representative diagram of canopy heights of the *A. germinans* trees found in each ecosystem type were then calculated and illustrated using the “mangroveStructure” package (Araújo and Shideler 2019) in RStudio (Version 2024.12.0+467).

#### 2.2.2 The Use of Allometric Equations to Determine Organ Weights of *A. germinans*

We applied the following established allometric equations developed by Komiyama et al. (2002) and Komiyama et al. (2005) to determine the specific weights (kg) of various sections of *A. germinans* trees found in each ecosystem type:

(1) $$\text{Trunk weight}\;\left(W_{T}\right)=0.0696\;\rho \;{\left(D_{2}H\right)}^{0.931}$$

(2) $$\text{Aboveground weight}\;\left(W_{\mathrm{AG}}\right)=0.251\rho D^{2.46}$$

(3) $$\text{Root weight}\;\left(W_{R}\right)=0.199\rho^{0.899}D^{2.22}$$

where *D* = diameter at breast height (DBH) (cm), *H* = height of the *A. germinans* tree (m), and *ρ* = specific density/gravity of *A. germinans* wood—0.64 g/cm^3^ (Virgulino‐Júnior et al. 2019).

Furthermore, to determine the trunk weights, aboveground weights, and root weights of *A. germinans* trees per ha, we utilized the following formula adopted by Kumarathunge (2011) and utilized by Dookie et al. (2023a; 2022a);

(4) $$\text{Tree organ weight}\;\left(\mathrm{kg}/\mathrm{ha}\right)=\frac{\text{Total weight of specific tree organ sampled along transect line}}{\pi \times {\left(\text{Radius of}\;\mathrm{one}\;\text{sampling point}\right)}^{2}\times \text{Total number of sampling points}\;}\times 10000$$

### 2.3 Data Analysis

The datasets were analyzed using various statistical tests, with a significance level of *p* < 0.05, utilizing Microsoft Excel, R (version 4.3.2), and R Studio programming software (Version 2024.12.0+467). To increase normality in the distribution of our datasets, all values obtained were subjected to log10 transformations. The Shapiro–Wilk test of normality (*W*) was then conducted, and reported values indicated that the datasets for trunk weight (*W* = 0.95758, *p* = 2.036e‐12), aboveground weight (*W* = 0.96044, *p* = 6.672e‐12), and root weight (*W* = 0.96044, *p* = 6.672e‐12) failed to confirm a normal distribution. As such, non‐parametric tests were employed on all datasets.

The following statistical analyses were utilized based on our objectives of the study. First, to quantify the trunk, aboveground, and root weights of *A. germinans* trees, we utilized several descriptive statistics such as the mean, standard error, and range to highlight existing values. To assess the variance in the means of each tree organ weight found within each ecosystem type, we conducted a series of Kruskal–Wallis tests using the R package “stats” (R Core Team 2013). Post‐hoc analyses using Dunn's test of multiple comparisons were subsequently conducted using the “dunn.Test” package (Dinno 2015) to determine which of the three mangrove ecosystem types had the greatest differences in their trunk, root, and aboveground weights. Second, to quantify the strength of correlations between the aboveground, trunk, and root weights in each ecosystem type, we generated Spearman rank correlation coefficients. Lastly, we conducted a multiple regression analysis using a generalized linear model (GLM) to determine the strength of the relationship between the independent variable “tree organ weight” and the dependent variable “location.” Both correlations and multiple regression analyses were computed using the “stats” package in R (R Core Team 2013).

## 3 Results

### 3.1 Description of *A. germinans* Trees

**FIGURE 3:**
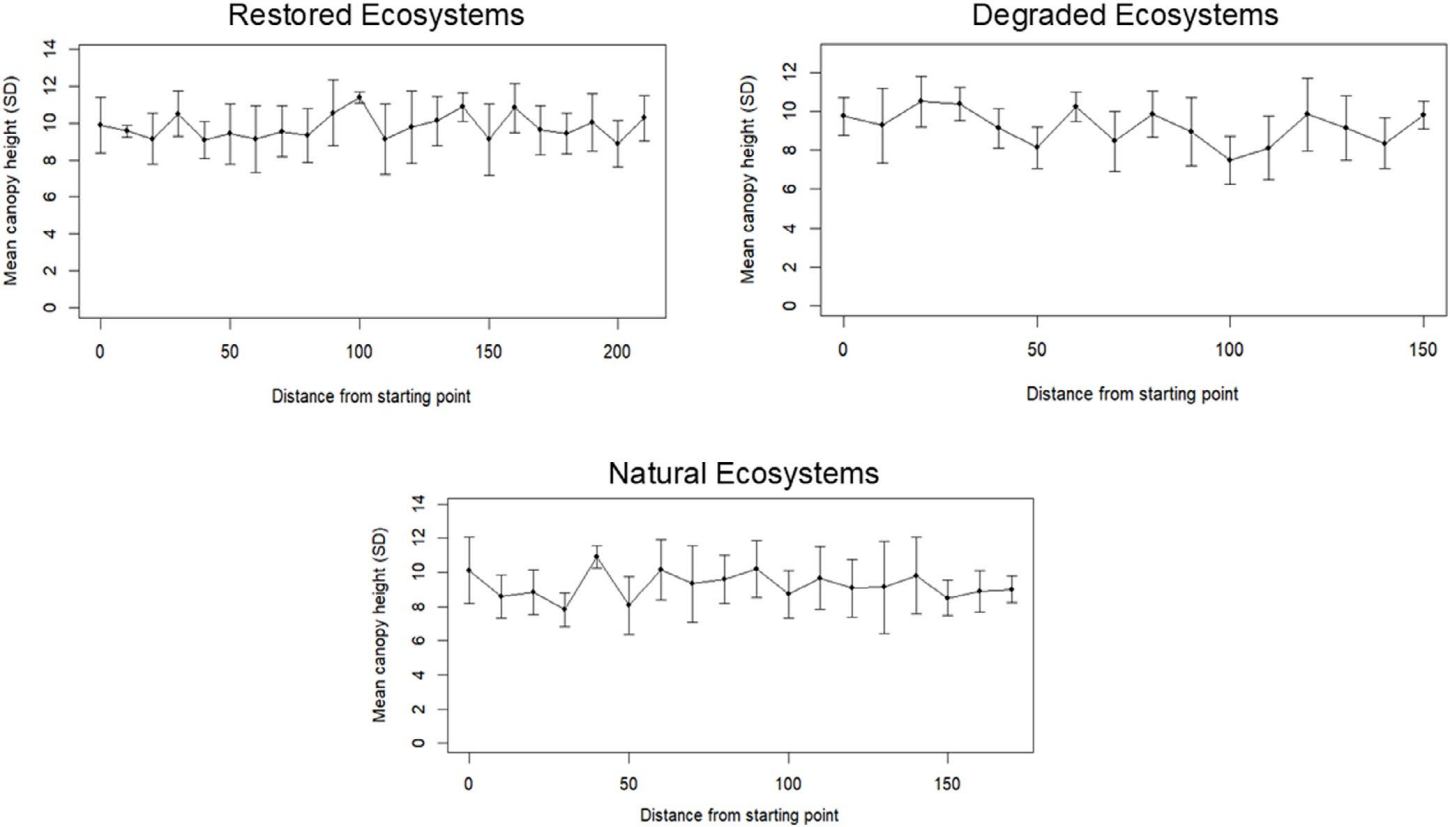
General representation of the mean canopy heights of *A. germinans* trees found within each ecosystem type.

The transect lines established within each ecosystem type recorded a total of 625 *A. germinans* trees, with the highest count observed in the natural ecosystems of the study (Table 2). Trees in natural ecosystems were categorized into four diameter classes: > 5–10, > 10–20, > 20–30, and > 30–40 cm. In contrast, trees in degraded and restored ecosystems were limited to the > 5–10 and > 10–20 cm diameter classes. The restored ecosystems exhibited taller trees (6.81–12.56 m) (Figure 3) while trees within the natural ecosystems displayed thicker trunks (8.66–32.72 cm), contributing to the largest overall basal area (14.09–24.81 m^2^/ha) observed in this study. However, the degraded ecosystems exhibited the lowest tree counts, with basal areas ranging from 2.15 to 4.62 m^2^/ha, in contrast to the natural and restored ecosystem types (Table 2).

**TABLE 2:** Dendrometric measurements of *A. germinans* trees in the natural, degraded, and restored mangrove ecosystems.

Ecosystem classification type	Stage of forest	Age of forest	Number of trees sampled	DBH class detected (cm)	Estimated tree density (individuals/ha)	Range of values		
						Stand basal area (BA) (m^2^/ha)	DBH (cm)	Height (m)
Natural	Secondary	> 15 years	250	5–10	84.20	14.09–24.81	8.66–9.07	7.28–11.43
				10–20	1133.55		14.92–19.33	7.18–12.50
				20–30	1152.50		22.71–25.56	7.40–12.49
				30–40	143.64		32.44–32.72	8.00–10.66
Degraded	Secondary	> 15 years	147	5–10	1414.68	2.15–4.62	7.83–8.47	5.73–11.44
				10–20	1232.93		12.47–14.31	6.03–11.46
Restored	Primary	< 15 years	228	5–10	4552.36	4.21–8.47	7.72–9.42	6.81–9.87
				10–20	3423.70		10.63–13.63	11.07–12.56

### 3.2 Trunk Weight ( *W*_T_ )

Our results highlighted a direct proportional relationship between the weight of the trunk and the diameter at breast height (DBH) of *A. germinans* trees. Natural ecosystems exhibited the highest trunk weights, ranging from 23.49 to 250.60 kg, followed by degraded ecosystems with weights between 18.77 and 44.26 kg, and restored ecosystems, which ranged from 18.42 to 42.85 kg (Table 3). The Kruskal–Wallis test indicated significant differences in the mean trunk weights of *A. germinans* trees across the three ecosystem types (*χ* ^2^ = 378.84, df = 2, *p* < 2.2e‐16). Additionally, Dunn's test of multiple comparisons indicated that the trunk weights of *A. germinans* trees from natural ecosystems were significantly different from those of the other two ecosystem types (*p* < 0.05).

**TABLE 3:** Average respective weights for *A. germinans* trees sampled in each ecosystem type by diameter class (mean ± SE).

Ecosystem classification type	DBH class detected (cm)	Mean ± SE		
		Trunk weight (*W*_T_) (kg)	Aboveground weight (*W*_AG_) (kg)	Root weight (*W*_R_) (kg)
Natural	5–10	23.49 ± 2.00	38.27 ± 2.93	18.59 ± 1.29
	10–20	73.46 ± 2.31	170.72 ± 5.74	71.40 ± 2.19
	20–30	149.78 ± 3.55	437.93 ± 12.50	167.11 ± 4.31
	30–40	250.60 ± 8.25	897.80 ± 16.55	320.82 ± 5.35
Degraded	5–10	18.77 ± 0.69	31.92 ± 1.39	15.68 ± 0.62
	10–20	44.26 ± 2.02	102.34 ± 4.99	44.96 ± 1.97
Restored	5–10	18.42 ± 0.48	34.39 ± 0.94	17.36 ± 0.46
	10–20	42.85 ± 1.73	94.35 ± 4.11	48.32 ± 2.09

### 3.3 Aboveground Weight ( *W*_AG_ )

Our findings indicated a direct proportional relationship between the aboveground weight of trees and their DBH, similar to the trunk weights observed in *A. germinans* trees. Natural ecosystems exhibited higher *W*_AG_ values (38.27–897.80 kg), especially within the 30–40 cm DBH class, in contrast to degraded ecosystems (31.92–102.34 kg) and restored ecosystems (34.39–94.35 kg) (Table 3). The Kruskal–Wallis test indicated significant differences in the mean aboveground weights of *A. germinans* trees across the three ecosystem types (*χ* ^2^ = 369.92, df = 2, *p* < 2.2e‐16). Furthermore, Dunn's test of multiple comparisons indicated that the aboveground weights of *A. germinans* trees in natural ecosystems were significantly different from those in the other two ecosystem types (*p* < 0.05).

### 3.4 Root Weight ( *W*_R_ )

Similar to *W*_T_ and *W*_AG_, a direct proportional relationship also existed between the root weights and the DBH of *A. germinans* trees. The natural ecosystems reported greater root weights (18.59–320.82 kg), particularly in the 30–40 cm DBH class, followed by the restored ecosystems (17.36–48.32 kg), and then the degraded ecosystems (15.68–44.96 kg) (Table 3). The Kruskal–Wallis test reported significant differences in the mean root weights of *A. germinans* trees found within the three ecosystem types (*χ* ^2^ = 360.57, df = 2, *p* < 2.2e‐16). Furthermore, Dunn's test of multiple comparisons reported that the root weights of *A. germinans* trees from the natural ecosystems significantly differed from the other two ecosystem types (*p* < 0.05).

### 3.5 Weight of Tree Organs Per Ecosystem Type

**FIGURE 4:**
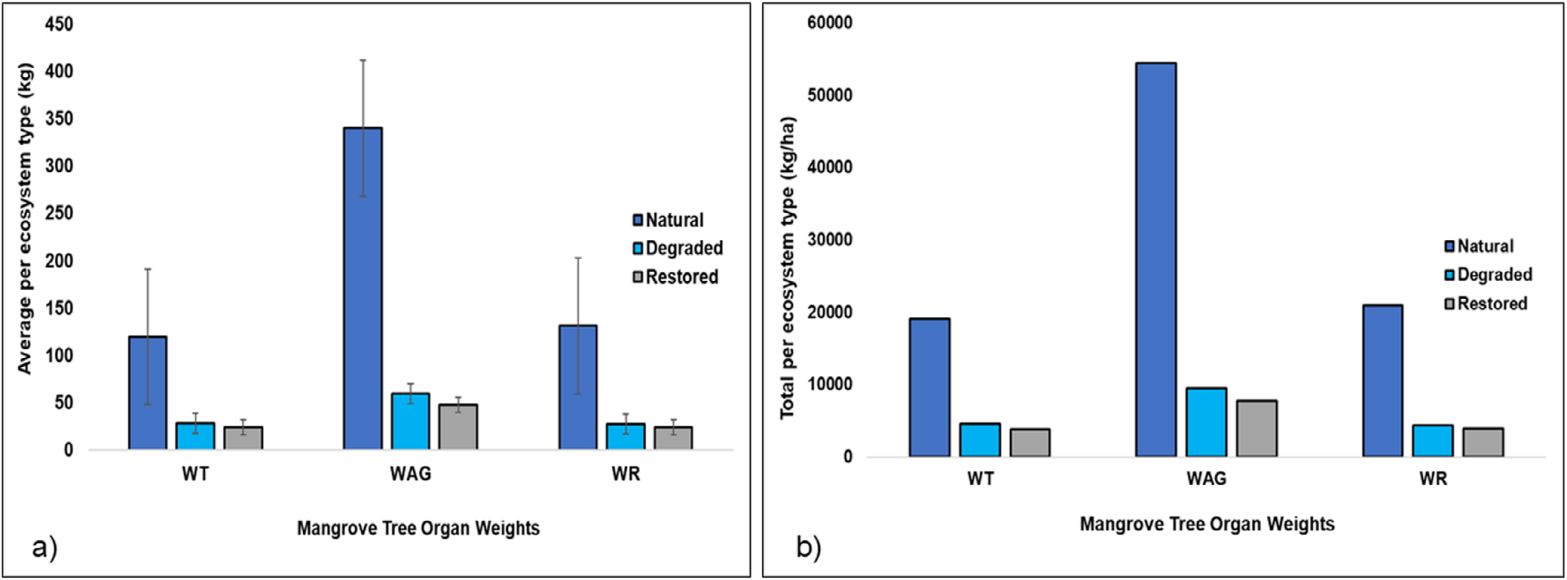
Overall mangrove tree organ weights per ecosystem type in (a) kg (mean ± SE) and (b) total per hectare (kg/ha). NB: WAG, aboveground weight; WR, root weight; WT, trunk weight.

The trees in the natural ecosystems exhibited higher average aboveground, trunk, and root weights when compared to the restored and degraded ecosystems (Figure 4a). This was also apparent when calculations were conducted on a per‐hectare basis. Our calculations indicated that the natural ecosystems exhibited higher total *W*_T_ (19127.08 kg/ha), *W*_AG_ (54396.24 kg/ha), and *W*_R_ (20984.44 kg/ha) compared to restored ecosystems (*W*_T_ = 3872.03 kg/ha, *W*_AG_ = 7731.91 kg/ha, *W*_R_ = 3933.54 kg/ha) and degraded ecosystems (*W*_T_ = 4543.26 kg/ha, *W*_AG_ = 9498.95 kg/ha, *W*_R_ = 4334.58 kg/ha) (Figure 4b).

### 3.6 Correlation Between *W*_T_ , *W*_AG_ , and *W*_R_ of *A. germinans* Trees

The Spearman rank correlation coefficients (*r*_s_) tabulated for the weights of different tree organs [*W*_T_~*W*_AG_], [*W*_T_~*W*_R_], and [*W*_R_~*W*_AG_] revealed strong positive correlations within all three ecosystem types (*p* < 0.05, *r*_s_ > 0.05). The strongest positive correlations between *W*_T_, *W*_AG_, and *W*_R_ were seen in the natural ecosystems (0.97 < *r*_s_ < 1.0), followed by the degraded ecosystems (0.96 < *r*_s_ < 1.0), and then the restored ecosystems (0.95 < *r*_s_ < 1.0).

### 3.7 Multiple Regression Analysis (GLM)— *A. germinans* Tree Organ Weights and Ecosystem Type

A multiple regression analysis using a GLM model was conducted to determine the strength of the relationship between the weights of the *A. germinans* tree organs and their respective ecosystem types. The results and estimates of the model (shown in Table 4) provide some evidence which indicates a significant relationship between “mangrove tree organ weights” (*W*_T_ + *W*_AG_ + *W*_R_) and “ecosystem type” (ET) in the degraded (*p* = 3.81e‐09) and natural ecosystems (*p* < 2e‐16), but not the restored ecosystems (*p* = 0.439). The model equation shown below indicates that with every unit rise in the intercept, all other units will show significant increases accordingly with the exception of the restored ecosystems.

(5) $$\begin{array}{c} \left[W_{T}+W_{\mathrm{AG}}+W_{R}\sim \mathrm{ET}\right]=\left(\text{Intercept}\right)\;114.94\\ +\mathrm{ET}\;|\text{Natural}|\;475.21-\mathrm{ET}\;|\text{Restored}|\;19.07 \end{array}$$

**TABLE 4:** Summary of multiple regression analysis using a generalized linear model—mangrove tree organ weights versus ecosystem type.

Coefficients	Estimate	Std. error	*t*‐value	Pr (>|t|)
Intercept	114.94	19.23	5.98	**3.81e‐09**
Ecosystem type: natural	475.21	24.22	19.62	**< 2e‐16**
Ecosystem type: restored	−19.07	24.62	−0.77	0.439
Null deviance	68,922,348 on 622 degrees of freedom			
Residual deviance	33,462,009 on 620 degrees of freedom			
AIC	8561.30			
Number of Fisher score iterations	2			

*Note:* Figures in bold type indicate that the result is significant (*p* < 0.05).

## 4 Discussion of Findings

Our findings indicated significant differences between the mangrove tree organ weights found in the different types of mangrove ecosystems, providing direct evidence to formally reject our null hypothesis in favor of the alternative. The weights and biomass accumulation in *A. germinans* tree organs were similar to those of Fromard et al. (1998), Virgulino‐Júnior et al. (2019), and Ávila‐Acosta et al. (2024) for *A. germinans* stands in French Guiana, Brazil, and Mexico, respectively. Our findings also indicated a correlation between tree weights and ecosystem types along the Guyana coastline, revealing that natural ecosystems exhibited trees with greater weights, followed by degraded ecosystems, and then restored ecosystems. Several factors contribute to these differences; however, two primary considerations are the age (maturity) of the trees and the degree of disturbances within the ecosystems (Maia and Coutinho 2012).

The natural ecosystems examined in this study exhibited shorter trees with thicker trunks, leading to larger DBH values in low‐disturbance environments. Low‐disturbance levels in mangrove ecosystems typically promote enhanced growth and a more diverse ecosystem by creating optimal circumstances for seedling establishment, root development, and overall plant vitality, while minimizing disruption to natural processes. Conversely, higher disturbance levels can substantially impede mangrove growth and diminish biodiversity (Akhtar and Tsuyuzaki 2024; Zimmer et al. 2022). In a mature mangrove forest, an individual tree's total weight can reach several tons due to the biomass collected in its leaves, stems, branches, and roots. As trees mature, they tend to devote more biomass to their stems and branches and less to their foliage. Their roots also develop to balance the distribution of resources, causing them to gain weight over time as biomass accumulates (Poorter et al. 2011). The accumulation in old trees is significant, as even‐aged forest stands exhibit reduced height, diameter, and volume growth in older tree ages, leading to a sigmoid‐shaped growth curve. Tree growth decline over time is linked to fluctuations in the availability of essential resources (light, nutrients, water), alterations in the balance between photosynthesis and respiration, heightened hydraulic resistance, reduced nutrient availability, or genetic modifications (Köhl et al. 2017). Trees can increase root biomass allocation to optimize the uptake of water and nutrients in nutrient‐limited, dry conditions (Wang et al. 2023). Mangroves may allocate significant biomass to their roots to sustain a bottom‐heavy tree form or a small proportion of top biomass to root biomass, aligning with our findings. This phenomenon can create unique conditions for ecosystem processes in root zones due to anaerobic conditions (Komiyama et al. 2008).

A significant proportionality is observed between aboveground, trunk, and root weights in mangroves, as demonstrated by the correlations and regression values in our study. Younger trees in restored ecosystems demonstrate distinct biomass allocation patterns, emphasizing stems, branches, and leaves to promote height and canopy development. This aids in the resource acquisition process, including light and water. Biomass allocation in the face of competition can influence its corresponding exponents (Zhang et al. 2011). Plant competition for resources such as light and nutrients may influence biomass allocation between root–shoot and stem–branch structures (Tang et al. 2022).

In light‐limited communities, such as dense forests, there is a trade‐off in biomass allocation between stems that allow a plant to overtop and shade neighbors with branches and supporting leaves, which enhance total light interception area (Zhang et al. 2012). Plants in the restored ecosystems examined in our study may adopt either the “height (escape)” strategy or the “radial (fight)” strategy, leading to different biomass allocation patterns. Our study demonstrated that *A. germinans* trees present in the restored ecosystems employed a “height (escape)” strategy, leading to increased height and reduced trunk diameter (smaller DBH values). This phenomenon may result from the intervention from humans in forest restoration, which has affected the spacing between seedlings and addressed challenges such as poor soil quality, invasive organisms, and disrupted ecological equilibrium. This enhanced the conditions for tree growth by improving soil nutrients, increasing water availability, and minimizing competition from undesirable plants, thereby facilitating the thriving and reestablishment of native species and promoting a healthier ecosystem.

Trees in degraded ecosystems exhibited low density and minimal basal area with below‐average heights and DBH values. The reduction in tree weight poses a critical challenge in degraded ecosystems, as the ability of these systems to store and retain carbon in forested regions is largely affected by the ongoing loss of trees (Dookie et al. 2024a). Disturbances in mangrove ecosystems significantly influence biomass allocation and weight, prompting mangroves to redirect resource allocations toward root development for stability and regeneration in response to human and environmental stressors (Wong et al. 2024). This frequently results in a higher root‐to‐shoot ratio, which may adversely affect the general condition and nutrient storage capacity of mangrove forests. Human activities such as development along the coast, aquaculture, pollution, and logging can disrupt mangrove ecosystems, leading to alterations in biomass allocation as a result of modified sedimentary dynamics and diminished canopy cover (Nwobi and Williams 2021; Castañeda‐Moya et al. 2013).

The cycling of essential nutrients is fundamental to mangrove productivity and biomass distribution. Biogeochemical cycles and forest productivity may be disrupted by disturbances including deforestation, alterations in hydrology from reservoirs, land‐use changes, a higher intensity and frequency of storms, rising temperatures, elevated atmospheric CO_2_ levels, and rising sea levels (Alongi 2018). The high mortality rates in degraded ecosystems adversely affect the allocation of biomass and the weights of mangrove tree organs. This ultimately results in trees appearing “starved” and “stunted,” making them more susceptible to soil erosion, strong winds, and rising tides (Anton et al. 2020).

Mangrove forests exhibit significant trends in biomass allocation and tree weight dynamics, contributing to their efficiency as carbon sinks in tropical ecosystems. The increase in atmospheric carbon dioxide concentration, along with major shifts in the environment, sea level rise, and global warming, is expected to severely affect the cycling of carbon through mangrove ecosystems (Gu et al. 2022). While our study was conducted on a short‐term basis, our findings indicate that the natural mangrove ecosystems showcase an affinity toward greater net primary productivity (NPP) while the degraded ecosystems indicated low NPP due to higher disturbances and tree mortality. The NPP of restored ecosystems may increase over time as the forest achieves stability through complete recovery. The depletion of mangroves due to deforestation, conversion to industrial zones or aquaculture, and changes in drainage systems has substantial effects on carbon cycling. These activities frequently lead to an increased release of atmospheric greenhouse gases, such as carbon dioxide, resulting in a transition of mangroves from carbon sinks to carbon sources, thereby diminishing their NPP over time (Dookie et al. 2024a). We predict that NPP will vary among different ecosystem types based on the nature and severity of disturbances present within each ecosystem. For instance, periodic flushing from river discharge and/or tidal inundation enhances nutrient availability because mature mangroves typically have nitrogen and phosphorus restrictions (Chatting et al. 2024). Moreover, enhanced precipitation diminishes soil salinity, thereby fostering increased productivity (Poungparn et al. 2020). Conversely, elevated temperatures and rising sea levels may alter inundation periods and durations, likely leading to increased tree mortality and a substantial decline in NPP in degraded or recovering ecosystems (Ward et al. 2016).

This necessitates a deeper understanding of sustainable forestry management in the conservation and restoration of mangrove ecosystems. The assessment of mangrove forest status is crucial for effective conservation planning and management. Management of mangrove biomass focuses on conservation strategies, including the reestablishment of protected areas, restoration initiatives to plant new mangroves in degraded regions, health monitoring of existing mangrove forests via surveys and satellite imagery, and the implementation of sustainable practices to reduce human impacts, such as regulating coastal development and managing resource extraction within mangrove ecosystems (Schmitt and Duke 2015). We believe that conserving existing mangrove forests is often more effective than replanting forests. As such, involving local communities along the Guyana coastline in sustainable mangrove management may be an effective way of maintaining and enhancing the productivity and functionality of mangrove forests. This facilitates ongoing livelihoods for local populations, enhances the evaluation and management of natural resources, and underscores the relationship between mangroves and society in addressing climate change.

## 5 Conclusion

Our study demonstrated significant differences in the aboveground, trunk, and root weights (kg/ha) of *Avicennia germinans* across various ecosystem types, particularly within natural ecosystems, thereby rejecting the null hypothesis. The natural ecosystems exhibited trees with higher overall organ weights, followed by degraded and restored ecosystems, attributable to their greater DBH values. The positive correlation coefficients and regression values provided compelling evidence supporting the association between biomass allocation via tree organ weights and ecosystem types. The results of our investigation support the notion that disturbance compositions within an ecosystem can affect the net primary productivity of mangrove forests over time, which may have an impact on their future biomass allocation and carbon storage capabilities. As a result, active conservation of existing mangrove forests is critical to their future production and functionality, as well as their broader significance within biogeochemical cycles and role in climate change mitigation.

## Conflicts of Interest

The authors declare no conflicts of interest.

## Data Availability

The data that support the findings of this study are openly available in Harvard Dataverse Repository at “Replication Data for: *Avicennia germinans* Tree Weights,” https://doi.org/10.7910/DVN/AUIRP1, V1.
